# 

**Published:** 1905-07-01

**Authors:** 


### NOTICE TO CORRESPONDENTS.

All MSS., Letters, Books for Review, and other matters intended for the Editor should be addressed The Editor, "The Hospital," The Hospital Buildings, 28 & 29 Southampton Street, Strand, W.C.

The Editor cannot undertake to return rejected MS., even when accompanied by stamped directed envelope.

**Notice to Advertisers and Subscribers.**

All Advertisements, Orders for Copies of The Hospital, and Business Communications should be addressed to **THE MANAGER (Not to the Editor),** The Hospital, 28 & 29 Southampton Street, Strand, London, W.C.

The Cost of Subscription, post free, is at follows:—Per week, 3½d.; per uarter, 3s. 3d.; per half-year, 6s. 6d.; per annum, 13s. Foreign and olonial:—Per annum, 19s. 6d., payable, in all cases, in advance.

**NOTICE.—Vol. XXXVII. of "The Hospital" October 1904 to March 1905), in handsome cloth binding, will shortly be on sale at the office of this Journal, price 10s. 6d. Cases for binding the Numbers contained in this Volume 2s. Index to Vol. XXXVII., 3½d., post free.**

**The Monthly Part for July is now ready. Price 1s., by post, 1s. 3d.**

### MEDICAL APPOINTMENTS.

**Ash, R. V. C.**, M.B., B.S.Edin., Lecturer on Anatomy, Physiology, and Hygiene at the Trust College of Hygiene and Medical Inspector of Schools, Dunfermline. **Cook, F. E.**, M.R.C.S., L.R.C.P.Lond., Medical Officer to attend to the Destitute Poor and Aborigines within the District of Millicent. **Crawford, A. R.**, M.B., Ch.B.,N.Z., Junior House Surgeon to the Dunedin Hospital, New Zealand. **Dyer, C. Harold**, M.D.Aberd., re-appointed Medical Officer of Health to the Cleckheaton Urban District Council. **Fox, Ida E.**, M.B., B.S.Durh., Resident Medical Officer, Sherwood Forest Sanatorium, Mansfield, Notts. **Gow, William Baxter**, M.D , M.S.Edin., Medical Superintendent of the Lunatic Asylum at Christchurch, New Zealand. **Macgilvray, W. Jackson Hooker**, L.R.C.P., L.R.C.S.Edin., etc., Medical Referee, Royal Liver Friendly Society, for Higher Broughton, Manchester, and District. **Macmillan, A. R.**, M.B., Ch.M.Edin., Medical Officer to attend to the Destitute Poor and Aborigines within the District of Naracoorte. **McMillan, J.**, M.B., B.S.Glasg., Certifying Surgeon under the Factory and Workshop Act for the Shotts District of the county of Lanark. **Wethered, F. J.**, M.D.Lond., F.R.C.P., Physician to the Hospital for Consumption and Diseases of the Chest, Brompton.

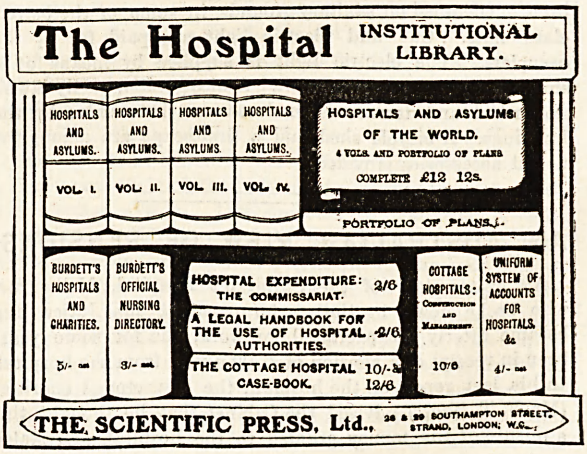

A SELECTION OF **Nursing Manuals** *Published by* THE SCIENTIFIC PRESS, Ltd., 28 & 29 Southampton St., Strand, London, W.C.

**AT 3/6**

**NURSING: ITS THEORY AND PRACTICE.**
By Dr. Percy G. Lewis.

**SURGICAL WARD-WORK AND NURSING.** With particulars of Instruments used in various operations. By Alexander Miles, M.D. 3/10 post free.

**OPHTHALMIC NURSING.**
By Sydney Stephenson, M.B. 3/10 post free.

**DIET IN SICKNESS AND IN HEALTH.**
By Mrs. Ernest Hart.

**OUTLINES OF INSANITY.**
By Francis H. Walmesley, M.D. Popular Edition 1/6.

**FIRST AID TO THE INJURED AND MANAGEMENT OF THE SICK.** By E. J. Lawless, M.D.

**AT 2/6**

**ELEMENTARY TREATISE ON THE LIGHT TREATMENT FOR NURSES.** By James H. Sequeira, M.D. Lond. 2/9 post free.

**ELEMENTARY ANATOMY & SURGERY FOR NURSES.**
By Wm. McAdam Eccles, M.S., M.B.

**HOSPITAL SISTERS AND THEIR DUTIES.**
By Eva C. E. Lückes.

**NURSING IN DISEASES OF THE THROAT, NOSE, AND EAR.** By P. Macleod Yearsley, F.R.C.S.

**SPINAL CURVATURE AND AWKWARD DEPORTMENT.**
By S. G. Muller. English Edition by Richard Greene, F.R.C.P.

**AT 1/=**

**THE MODERN NURSING OF CONSUMPTION.**
By Dr. Jane Walker. 1/2 post free.

**FEVERS AND INFECTIOUS DISEASES.**
By Wm. Harding, M.D.

**MIDWIVES' POCKET BOOK.**
By Honnor Morten.

**GYNÆCOLOGICAL NURSING.**
By Hawkins Ambler, F.R.C.S.

**NOTES ON PHARMACY & DISPENSING FOR NURSES.**
By C. J. S. Thomson.

**THE EXAMINATION OF THE URINE.**
By Dr. J. K. Watson. 1/1 post free.

**AT 6d.**

**ON PREPARATION FOR OPERATION IN PRIVATE HOUSES.** By Stanmore Bishop, F.R.C.S.

**MODERN NURSING IN PRIVATE PRACTICE.**
By Sir William Bennett. 7d. post free.

**"SIMPLEX" DISTRICT NURSES' CASE-BOOK.**
Containing space for 50 cases, ruled and printed. 7d. post free.

*NEW CATALOGUE OF NURSING MANUALS POST FREE ON APPLICATION.*

**THE SCIENTIFIC PRESS, LTD.,** Publishers and Booksellers of all kinds of Nursing Text-books, Case-books, Charts, &c.

**28 & 29 Southampton St., Strand, London, W.C.**

### The NURSING MIRROR.

**The Nurse's Own Paper.**
**Price ONE PENNY Weekly.**
Of all Booksellers and Newsagents, or posted direct from the Office.
*Write for Subscription Rates to The Manager,*
THE NURSING MIRROR, The Hospital Building, 28 & 29 Southampton Street, Strand, London, W.C.

